# Anent the Fever Situation in San Antonio

**Published:** 1903-12

**Authors:** L. L. Shropshire


### Anent the Fever Situation in San Antonio.

BY DR. L. L. SHROPSHIRE.

*Editor Texas Medical Journal:*

"Truth is violated by falsehood, and may be equally outraged by silence."

It is said that patience sometimes ceases to be a virtue. That this time has arisen in our city goes without saying. The situation has been so grossly misrepresented that I feel that I am recreant to my duty to the profession of the State in keeping silent any longer. That we have yellow fever in San Antonio no sane man can gainsay, but that the true situation is known I doubt seriously.

The disease was early recognized by some of our leading practitioners, and when our most competent and efficient State health officer arrived upon the scene with his world renowned assistant in the person of that noble warrior, the late lamented Dr. Murray, a large majority, at least 90 per cent, of our profession laid aside prejudice and self-aggrandizement, came nobly to his assistance, acknowledged the situation and proffered their services in every way possible to stamp out the malady. But just at this juncture a meager few of our number—not more than five or six—arrayed themselves against the State health officer, the United States marine surgeons and more than 90 per cent of the most respectable element of the local profession, and took the stand that this prevailing disease was anything from the mildest forms of dengue to the most virulent form of malignant malaria.

In the first place, they took their stand without autopsies or requesting the courtesy to be taken to see a case which had been diagnosed yellow fever; but after a good deal of urging they at last saw one or two cases and autopsies; and then came the deluge of diagnotic acumen thrust upon an unsophisticated public through the daily press, that would turn old Esculapius over in his grave. It was called "acute yellow atrophy of the liver," "malaria fulminated with dengue," "pernicious malaria," "yellow jaundice," "dengue fever," "*nephritis of the kidneys*," and many other equally ridiculously named diseases. The wish being the father of the thought with the laity, they took up the ball and kept it rolling, talking and writing more nonsense about yellow fever than was ever heard before, and, I trust, will ever be heard again. When these sages of the malarial theory were pressed as to the microscopic examination of the blood, they would say that it "used to be a science to make a diagnosis of malaria, but now you fellows have to have a microscope and some of the blood to tell whether or not a patient has malarial fever." Another would pride himself that, he "never looked into one of the damn things and never expected to." We can pass up and excuse such ignorance of diagnosis except when it comes to defaming the fair name of our loved and honored city by publishing to the world that she is a hotbed of pernicious malarial fever with a death rate of nearly 50 per cent of those who are so unfortunate as to contract it.

That the climate is almost free from malaria in any form is a well known fact, and it has not been long since these very same men have publicly announced that we never have malaria in San Antonio. The inevitable result of this stand, the so-called "honor roll" of physicians consisting of five or six men has taken, has led most of the victims of the disease to send for one of these so-called "saviors of the people" who has treated them for malaria, dengue, etc., with the lamentable result that can best be told by the undertaker. Many people have had the disease in a mild form, for it certainly is a mild form, as the death rate at Laredo shows, and have failed and refused to send for physicians at all, therefore their cases have not been recorded. In fact, most of the cases which have been reported at all have not been reported until it was patent that some one would have to sign a death certificate very soon. The medical profession has been presented with the proposition of boycott and other dire disasters if they reported cases of yellow fever. We have been derided and ridiculed in the most amusing and outrageous manner by the people and the press. Men have dared us to show them black vomit, offering to drink all we can produce, and others have offered to sacrifice themselves upon the altar of fame by daring us to turn the so-called infecto-mosquitoes loose upon them, but as our statutes make no distinction between willfully murdering the sane and insane, we were compelled to decline the proffer. Those doctors who would be the first to resent the insult of being called unprofessional gentlemen, have aired themselves and their medical opinions in the lay press in the most ridiculous manner, so much so that many of our intelligent laymen have ridiculed their statements beyond measure, and have upbraided us because we did not do just what these gentlemen were anxiously expecting, answer their harangues through the same media. The profession of San Antonio is to be congratulated upon the fact that it has kept its head, and has not entered into fruitless and endless discussions of scientific subjects in the lay press. In these many newspaper interviews and articles, they have distorted and perverted the facts demonstrated upon autopsy, and have misled the public in many artful and designing ways. They have made it appear that one single infecto mosquito ("if it be the mosquito") has hovered over our city for weeks, and has descended at intervals of days, and at distance of miles, down upon one certain individual and has taken his flight to other remote parts, and infected another subject, and so on until he had reached all of the twenty-five or thirty cases reported. They have been prodded by the profession and many intelligent laymen about the use of the microscope, and the plasmodium until they have been compelled to shift anchor, and suddenly find an epidemic of acute yellow atrophy of the liver, a very uncommon disease; so much so that many physicians go through life without ever having the opportunity to see one case. They have told the public that the black vomit "was not black vomit at all, but simply hemorrhages of the stomach, which is a common occurrence in malaria and dengue." In giving their opinion upon a post-mortem when there was evidence of black vomit trickling from the corners of the mouth, they have been heard to say that "anybody could tell she was a snuff dipper." They have absolutelv and persistently denied the fact that it is necessary to have an enlarged spleen in pernicious malarial poisoning. They have charged the authorities with not screening these patients when they knew, if they knew anything at all, that the cases were not seen until after the fifth day of illness, and that it was not necessary at that late day. What these men could expect to accomplish by their persistent and unheard of policy it is hard to say.

Does it become medical men to array themselves against the national and State authorities and attempt to hamper and obstruct their work? Do they expect by their unwise course to crown themselves with temporary laurels, and by their action gain the applause and commendation of the business world, when they must know from what they have seen and from the surrounding circumstances that they may be mistaken, and that lives as precious and as valuable as their own, may be sacrificed by their action? No, as Lincoln has said, "they may fool all the people sometimes, and some of the people all the time, but they can not fool all the people all the time." After the governor's proclamation had been issued we had yellow fever *de jure* if not *de facto;* then would it not have been more professional and more patriotic for them to have laid aside their selfish motives, offered their hands to the authorities to assist in every way possible to stamp out the disease whether it be yellow fever, pernicious malaria or whatnot?

"Truth crushed to earth shall rise again,
The eternal years of God are her's,
But error, wounded, writhes in pain,
And dies among her worshipers."

After it is too late these very same men will seek and hide behind the greed of commercialism and say "we tried to save you, but alas! our efforts have been in vain, and now we must accept your condemnation for our illadvised bigotry and unpardonable ignorance." Fortunate indeed for them an all-wise Providence has blessed us with cold weather, which may check or stamp out the disease, but without this they would soon be seen in sack cloth and ashes importuning the forgiveness of an outraged public.

